# Data-Driven Surveillance Protocol for Patients at Risk for Peritoneal Recurrence of Primary Colon Cancer: Surveillance for Peritoneal Carcinomatosis †

**DOI:** 10.3390/jcm13082358

**Published:** 2024-04-18

**Authors:** Meloria A. Hoskins, Adam Finkelstein, Aisha Rashid, Olivia Ziegler, Marc M. Mankarious, Jorge V. Benavides, Colette R. Pameijer

**Affiliations:** 1 College of Medicine, Pennsylvania State University, 500 University Drive, Hershey, PA 17033, USA; mhoskins@pennstatehealth.psu.edu (M.A.H.); afinkelstein@pennstatehealth.psu.edu (A.F.); arashid@pennstatehealth.psu.edu (A.R.); 2 Department of Surgery, College of Medicine, Pennsylvania State University, 500 University Drive, Hershey, PA 17033, USA; oziegler@pennstatehealth.psu.edu (O.Z.); mmankarious@pennstatehealth.psu.edu (M.M.M.); 3 Division of Surgical Oncology, Department of Surgery, College of Medicine, Pennsylvania State University, 500 University Drive, Hershey, PA 17033, USA

**Keywords:** carcinomatosis, colorectal neoplasms, peritoneal neoplasms, recurrence

## Abstract

Peritoneal carcinomatosis (PC) is rarely discovered early due to low sensitivity of screening imaging and tumor markers, however, earlier identification may improve outcomes. This study assesses risk factors and time to recurrence of PC and implementation of a surveillance system. Patients with stage II–III colon adenocarcinoma undergoing curative colectomy between 2005–2022 were retrospectively reviewed at a single tertiary care institution. Patients were divided into three cohorts: no recurrence (NR), PC, and other types of recurrence (OTR). Baseline characteristics between cohorts were compared with univariate analysis. Overall survival and PC risk were assessed using multivariate analysis with Cox’s proportional-hazard modelling. 412 patients were included; 78.4% had NR, 7.8% had PC, and 13.8% had OTR. Patient demographics, comorbidities, tumor side, and histologic features were similar between cohorts. Patients with PC were more likely to have microscopic tumor perforation (25% vs. 8.8% vs. 6.8%, *p* = 0.002), margin involvement (25% vs. 8.8% vs. 4.6%, *p* < 0.001), lymphovascular invasion (56.2% vs. 33.3%, vs. 24.5%, *p* < 0.001), perineural invasion (28.1% vs. 15.8% vs. 11.5%, *p* = 0.026) compared to OTR or NR. Median time to PC after colectomy was 11 months. Tumor characteristics of stage II–III colon cancer define a high-risk profile for PC. An early surveillance program sensitive for peritoneal disease should be adopted for these patients.

## 1. Introduction

Peritoneal carcinomatosis (PC) is the third most common site of colon cancer metastases, following the liver and lung [1]. While the incidence of PC in patients with colon cancer varies between 4% to 18%, its actual incidence could be as high as 40% based on autopsy studies [2]. Although PC often has a poor prognosis with a median survival of approximately nine months, advances in cytoreductive surgery (CRS) and heated intraperitoneal chemotherapy (HIPEC), support an improvement in the five-year overall survival rate for patients with PC to a median of forty-one months [3].

To adequately identify and treat patients with PC, various studies aimed to identify risk factors that can aid in the early detection of PC [4]. In patients with locally advanced colon cancer, several risk factors such as age ≥ 60, female sex, T4 tumors, right-sided colon cancer, aggressive histopathologic features, or lymph node involvement have been identified [5,6]. Recent trials, including PROPHYLOCHIP and COLOPEC 2, have confirmed the risk of peritoneal recurrence in patients with high-risk features and support the use of second look surgery in early identification of PC-recurrence [7,8]. Despite these advances, there are no specific surveillance guidelines for patients with high-risk features for PC. The National Comprehensive Cancer Network (NCCN) and European Society of Medical Oncology (ESMO) recommend abdominal and chest CT surveillance every 6–12 months for 3–5 years, while the American Cancer Society (ACS) and the American Society of Clinical Oncology (ASCO) recommend CT every 12 months for 5 years [9,10].

The present study investigates high-risk features among patients with PC recurrence of colon cancer in a local population and analyzes the time to recurrence, aiming to inform a surveillance protocol to enable earlier detection and treatment of recurrence. We hypothesize that a set of high-risk features are associated with peritoneal recurrence and median time to recurrence can guide institutional surveillance protocol for early detection of PC.

## 2. Methods

Study Design and Patient Selection: This is a retrospective cohort study of adult patients with American Joint Committee on Cancer (AJCC) pathological stage at surgery of II or III colon adenocarcinoma who underwent curative colectomy at a single, tertiary-care institution from 2005 to 2022 located in Northeast United States. Patients with rectal cancer were excluded given the important differences in both the behavior and management of rectal cancer. Institutional Review Board approval was obtained (IRB#7806). Patients were stratified by type of recurrence: no recurrence (NR), peritoneal carcinomatosis (PC), and other type of recurrence (OTR) based on review of medical record during follow-up period. Patients with missing or incomplete data were excluded.

Variables: Medical records were reviewed for patient demographics including age at diagnosis, sex, race, and ethnicity. Patient past medical history including history of inflammatory bowel disease (IBD), polyposis syndromes, body mass index (BMI), and an unadjusted Charlson Comorbidity Index (CCI) was calculated based on documentation in the electronic medical record (EMR). Characteristics of the colon cancer included gross perforation or obstruction.

Pathology reports were reviewed and histopathological characteristics included T-stage, N-stage, lymphovascular invasion, perineural invasion, tumor deposits, mucinous component > 50%, microscopic tumor perforation, tumor grade, greatest tumor dimension, involvement of surgical margins, lymph node ratio (defined as number of positive nodes divided by number of nodes examined), and microsatellite instability.

Treatment details and outcomes included sequence of systemic therapy (neoadjuvant or adjuvant), surgical approach (minimally invasive including robotic or laparoscopic surgery or open surgical approach), blood transfusions, and time from surgery to recurrence.

Follow-up was calculated from date of primary colon resection to last clinical encounter. For patients with loss-to-follow-up, public obituaries were queried and dates of death, if applicable, were noted.

Statistical Analysis: Univariate analysis of cohorts was used to compare demographics, treatment, and tumor characteristics using Pearson’s chi-squared test for categorical variables and Wilcoxon rank sum test for continuous variables. Kaplan-Meier overall survival for the three cohorts and for disease-free survival in the PC or OTR cohorts was compared using the log-rank test. Multivariate analysis with Cox’s proportional hazard regression analysis was used to assess for independent predictors of overall survival and expressed as hazard ratios (HR) with 95% confidence intervals (CI). *p* values < 0.05 were considered statistically significant. Variables that were statistically significant in the univariate model or clinically significant were incorporated in the multivariable regression model and included type of recurrence, age at diagnosis, presence of polyposis, tumor laterality, use of neoadjuvant systemic chemotherapy, gross or microscopic tumor perforation, mucinous component > 50%, tumor grade, presence of positive surgical margins, lymphovascular invasion, perineural invasion, tumor deposits, lymph node ratio, tumor N-stage, and microsatellite instability. All statistical analyses were performed using R statistical software (Version 4.2.0).

## 3. Results

### 3.1. Patient and Disease Characteristics

A total of 412 patients were identified of whom 78.4% had NR, 7.8% had PC, and 13.8% had OTR (Table 1). Patients with PC were younger compared to patients with OTR or NR (62 vs. 63 vs. 69 years-old, respectively, *p* = 0.003). Demographic features such as sex, race, ethnicity, BMI, history of smoking, CCI, and history of IBD were similar between the cohorts. Patients with PC were more likely to have a history of polyposis (12.5% vs. 1.8% vs. 3.4%, *p* = 0.027) compared to patients with OTR and NR, respectively.

When evaluating pathologic characteristics, those with PC were more likely to have microscopic tumor perforation (25% vs. 8.8% vs. 6.8%, *p* = 0.002), margin involvement (25% vs. 8.8% vs. 4.6%, *p* < 0.001), lymphovascular invasion (56.2% vs. 33.3%, vs. 24.5%, *p* < 0.001), perineural invasion (28.1% vs. 15.8% vs. 11.5%, *p* = 0.026), T4 disease (71.9% vs. 35.1% vs. 19.5%, *p* < 0.001) and N2 disease (34.4% vs. 29.8% vs. 13.9%, *p* = 0.006) compared to patients with OTR or NR, respectively (Table 2). Patients with PC were more likely to have received adjuvant therapy (87.5% vs. 75.4% vs. 48.9%, *p* < 0.001). Overall, 229 patients received chemotherapy, 105 (45.8%) of whom received FOLFOX alone for a median of 6 cycles. Other common regimens included 21 patients who received capecitabine for a median of 6 cycles, 19 patients who received Xelox for a median of 8 cycles, 17 who received Xeloda for a median of 8 cycles, and 15 who received CAPOX for a median of 6 cycles.

Only 13 patients received neoadjuvant therapy prior to initial colectomy, with no differences in rates of neoadjuvant therapy observed between recurrence cohorts. The most common regimens were FOLFOX alone, or FOLFOX, FOLFOXIRI, or FOLFIRI in concert with Avastin.

### 3.2. Recurrence and Survival Analysis

**Figure 1:**
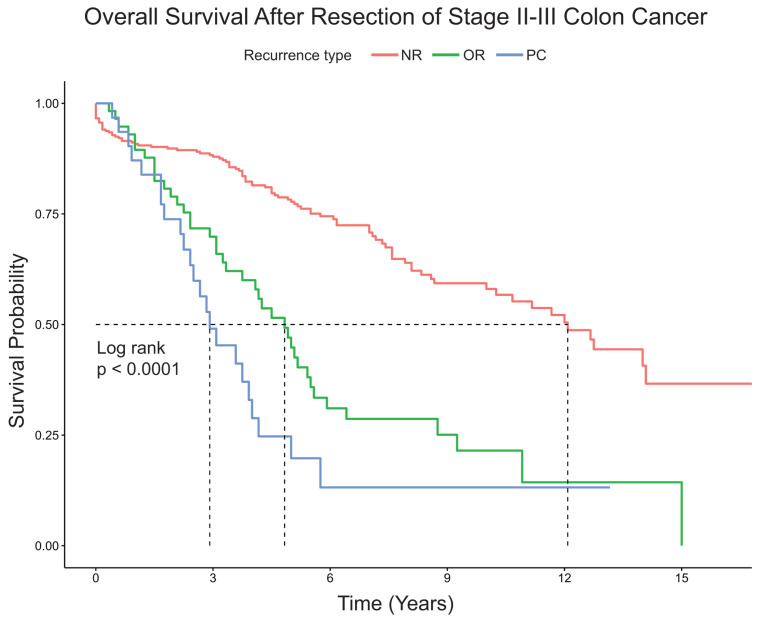
Kaplan Meier estimates for overall survival after resection of stage II–III colon cancer. Abbreviations: NR: No recurrence; OR: Other recurrence; PC: peritoneal carcinomatosis. Dotted line indicates median survival.

**Figure 2:**
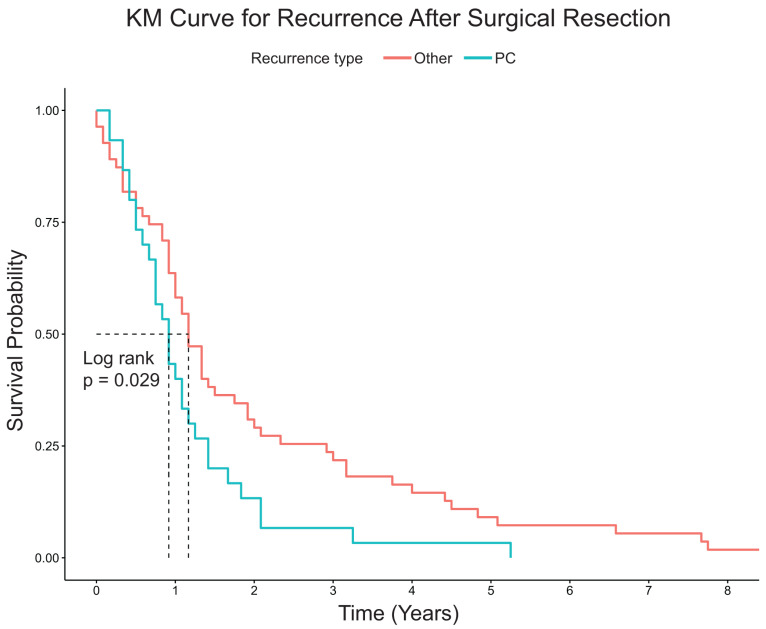
Kaplan Meier estimates for recurrence after surgical resection. Abbreviations: Other: Other recurrence; PC: peritoneal carcinomatosis. Dotted line indicates median survival.

Patients with PC had significantly shorter median overall survival compared to patients with OTR or NR (2.9 years vs. 4.8 years vs. 12.1 years, respectively, *p* < 0.0001) (Figure 1). For patients with PC or OTR, median time to recurrence from surgery for PC was shorter compared to patients with OTR (11 months vs. 14 months, *p* = 0.0029) (Figure 2). On multivariable Cox regression analysis, factors associated with increased mortality included OTR (HR 3.72 95%CI 2.30–6.01) and PC (HR4.79 95%CI 2.61–8.76) compared to NR (Table 3), but no other clinical or pathological features were significant.

## 4. Discussion

Patients with colon cancer at risk of developing PC in our cohort presented with T4 tumors, gross or macroscopic perforation, lymphovascular or perineural invasion, a positive resection margin and often N2 disease. This profile parallels other studies such as the COLOPEC trial which found that 21% of subjects with T4 or perforated colon cancers developed PC within three years of follow-up [11]. Mayanagi et al. determined that pathological T4 tumors and lymph node involvement were predictive of developing PC from stage II–III colon cancer [6]. Perineural and lymphovascular invasion are associated with PC [12,13] as is margin involvement [13]. Twenty-five percent of our patients with PC had mucinous tumors, although this was not significantly different from the OTR and NR patients. While mucinous type tumors have been associated with peritoneal recurrence in other series [12,14] it is possible that our patient numbers were too small to detect a difference. Advanced nodal disease as a predictor of PC is somewhat problematic, as nodal disease increases the stage of disease and the risk of metastasis overall. Features of perforation or T4 disease are more consistently associated with peritoneal recurrence compared to other sites of recurrence.

The median time to peritoneal recurrence is not well studied, but two recent trials of adjuvant HIPEC for high-risk patients indicate the risk and timing of recurrence. In the COLOPEC trial, patients with T4 colon cancers were randomized to undergo HIPEC in addition to standard systemic chemotherapy versus systemic chemotherapy alone, with diagnostic laparoscopy (DL) planned for all patients at 18 months. Of the 100 patients in the experimental group, 17 had already developed peritoneal metastasis and 16 of 102 patients in the control group had developed peritoneal metastases by 18 months, making them ineligible for DL. At the time of DL, two subjects in the experimental group and seven control group subjects were found to have peritoneal metastasis [15]. The PROPHYLOCHIP trial evaluated the benefit of second-look surgery and HIPEC at six months for patients with perforated colon cancers, peritoneal disease or ovarian metastasis at diagnosis. Subjects who were randomized to second-look laparotomy at six months after diagnosis were found to have peritoneal disease in 52% of cases (37/71), with a median Peritoneal Cancer Index (PCI) of 4 [14]. The median time to recurrence for our patients was 11 months, which correlates with the COLOPEC and PROPHYLOCHIP experience. Some early recurrences may in fact represent progression of peritoneal disease that was missed at the time of surgery, which accounts for some of the patients in both clinical trials [7,8]. The median survival of our patients with PC was only 2.9 years, compared to 4.2 years if they had OTR. Similarly, the three-year overall survival was 80% and 79% in the surveillance and HIPEC groups in PROPHYLOCHIP [14]. The number of patients treated with CRS/HIPEC in our study was too small to draw any conclusions or make any meaningful comparisons.

Current NCCN surveillance guidelines for stages II–III colon cancer include a history and physical exam and carcinoembryonic antigen (CEA) level every three to six months and CT scan of chest, abdomen and pelvis every six to twelve months for the first two years [9]. CEA elevation should prompt additional imaging or colonoscopy. Metastatic disease is assessed as resectable or unresectable, and systemic treatment options vary depending on mutation status of the tumor. The only algorithm that includes peritoneal metastasis is for dMMR/MSI-H synchronous metastatic disease, with the algorithm offering systemic treatment alone with surgical management of obstruction as needed. The “Principles of Surgery” section discusses management of liver and lung metastases only, with discussion of peritoneal disease reserved for the manuscript section. Here it is noted that overall survival outcomes are worse for patients with peritoneal metastases, the goal of treatment for most patients is palliative and consists of systemic therapy. Clinical trial results of CRS/HIPEC are discussed, but with the caveat that the approach is very controversial. No further specific recommendations for surveillance are made. In contrast, the subsequent section discusses resection of liver metastases in great detail, including early evaluation by a multidisciplinary team that includes a hepatic surgeon and patient selection criteria.

We propose additional surveillance guidelines for patients at risk of peritoneal recurrence. In addition to standard H&P, CEA and cross-sectional imaging, we recommend DL approximately 9–12 months after primary surgery. DL is safe [16] and typically an outpatient procedure. The subsequent management of peritoneal disease may include systemic chemotherapy and/or CRS with or without HIPEC. Second-look surgery is not a new recommendation [17] and is the focus of the COLOPEC 2 trial (NCT03413254) in which subjects with T4 colon cancer are randomized to DL at 6–9 months vs. DL at 6–9 months and a third DL at 18 months [8]. The prognosis for PC is poor despite CRS/HIPEC therapy once symptoms or imaging findings are present. Considering that preoperative CT scans may fail to visualize PC when the tumor nodules are small [18,19,20] surveillance with second-look surgery can fill this important gap in information. It is unknown whether early identification of peritoneal disease impacts outcomes, but the survival benefit from CRS/HIPEC is associated with completeness of cytoreduction (CC). CC is, in turn, related to burden of disease (PCI), which suggests that occult metastases are more amenable to complete resection and better outcomes. The five-year overall survival in patients with PC recurrence is only 10% in patients with a PCI above twenty compared to 49% in patients with a score under seven [21] reinforcing the benefit of early diagnosis of PC. The role of circulating tumor DNA (ctDNA) for early detection of peritoneal disease is unclear, but data suggests that ctDNA levels are significantly lower in patients with peritoneal only metastasis, with a ctDNA fraction (variant allelic fraction, VAF) of less than 1% compared to liver metastases, which have a VAF over 20% [22].

The present study has several limitations. As a retrospective cohort design, only correlation, not causation, could be established between primary colon cancer and metachronous PC. Additionally, the demographics of the study may lead to geographical bias. As PC was present in only 32 patients, multivariable logistic regression that accounts for multiple different patient variables and confounders could not be conducted. Furthermore, as our institution is a tertiary care center, referral bias may skew the findings of this study compared to a population-based studies.

## 5. Conclusions

Primary stage II–III colon cancer with PC is associated with lower overall survival and a shorter time to recurrence when compared to primary colon cancer with other types of recurrence. These findings emphasize the importance of implementing surveillance programs for patients with high-risk features for PC within the first year of surgical resection of the primary tumor. DL is safe and effective, although less invasive methods of surveillance should be developed to detect peritoneal recurrence.

## Figures and Tables

**Table 1:** Baseline characteristics. Abbreviations: BMI: Body Mass Index; CCI: Charlson Comorbidity Index; IBD: Inflammatory Bowel Disease.

	PC (*n* = 32)	OTR (*n* = 57)	NR (*n* = 323)	*p*-Value
Age, median (IQR)	62 (50.5–73.5)	63 (53–71)	69 (58–78)	0.003
Sex, *N* (%)				0.864
Male	15 (46.9)	26 (45.6)	159 (49.2)	
Race, *N* (%)				0.752
Black	2 (6.2)	2 (3.5)	8 (2.5)	
Other	1 (3.1)	3 (5.3)	12 (3.7)	
White	29 (90.6)	52 (91.2)	303 (93.8)	
Non-Hispanic, *N* (%)	32 (100.0)	57 (100.0)	317 (98.1)	0.432
BMI, median (IQR)	26.98 (22.95–33.11)	27.05 (24.40–31.91)	28.00 (23.99–32.98)	0.914
Smoking history, *N* (%)	12 (37.5)	20 (35.1)	131 (40.6)	0.716
CCI, median (IQR)	4 (3–6)	5 (4–6)	5 (4–6)	0.271
IBD	3 (9.4)	0 (0.0)	13 (4.0)	0.086
Polyposis, *N* (%)	4 (12.5)	1 (1.8)	11 (3.4)	0.027

**Table 2:** Comparison of disease characteristics and treatment between cohorts.

	PC (*n* = 32)	OTR (*n* = 57)	NR (*n* = 323)	*p*-Value
Surgery to Recurrence, months, median (IQR)	11.0 (6.3–16.50	14.0 (9.0–31.5)	NA	0.064
Neoadjuvant Systemic Therapy, *N* (%)	1 (3.1)	4 (7.0)	8 (2.5)	0.195
Adjuvant Systemic Therapy, *N* (%)	28 (87.5)	43 (75.4)	158 (48.9)	<0.001
Open Surgical Approach, *N* (%)	18 (56.2)	37 (64.9)	175 (54.5)	0.346
Bowel Diversion, *N* (%)	8 (25.0)	11 (19.3)	68 (21.1)	0.817
Blood Transfusion, *N* (%)	2 (6.2)	1 (1.8)	12 (3.7)	0.548
Right-sided tumor, *N* (%)	24 (75.0)	33 (57.9)	189 (58.5)	0.185
Obstruction, *N* (%)	8 (25.0)	9 (15.8)	42 (13.0)	0.171
Gross perforation, *N* (%)	8 (25.0)	6 (10.5)	17 (5.3)	<0.001
Microscopic tumor perforation, *N* (%)	8 (25.0)	5 (8.8)	22 (6.8)	0.002
Tumor Deposits, *N* (%)	12 (37.5)	14 (24.6)	59 (18.3)	0.027
Margins involved, *N* (%)	8 (25.0)	5 (8.8)	15 (4.6)	<0.001
Mucinous component > 50%, *N* (%)	8 (25.0)	8 (14.0)	57 (17.6)	0.428
Microsatellite instability, *N* (%)	4 (12.9)	15 (34.9)	44 (15.7)	0.007
Lymphovascular invasion, *N* (%)	18 (56.2)	19 (33.3)	79 (24.5)	<0.001
Perineural invasion, *N* (%)	9 (28.1)	9 (15.8)	37 (11.5)	0.026
Tumor Grade, *N* (%)				0.205
well	2 (6.2)	2 (3.5)	10 (3.1)	
moderate	22 (68.8)	43 (75.4)	270 (83.6)	
poor	2 (6.2)	2 (3.5)	16 (5.0)	
undifferentiated	2 (7.1)	1 (2.1)	14 (7.1)	
T stage, *N* (%)				<0.001
1	0 (0.0)	1 (1.8)	6 (1.9)	
2	0 (0.0)	1 (1.8)	13 (4.0)	
3	9 (28.1)	35 (61.4)	241 (74.6)	
4	23 (71.9)	20 (35.1)	63 (19.5)	
N stage, *N* (%)				0.006
0	9 (28.1)	20 (35.1)	166 (51.4)	
1	12 (37.5)	20 (35.1)	111 (34.4)	
2	11 (34.4)	17 (29.8)	45 (13.9)	
3	0 (0.0)	0 (0.0)	1 (0.3)	

**Table 3:** Cox regression analysis hazard ratios for overall survival.

Variable	HR	95% CI	*p*-Value
Type (no recurrence, reference)			
Other recurrence	3.72	(2.30, 6.01)	<0.001
PC	4.79	(2.61, 8.76)	<0.001
Age at Diagnosis	1.06	(1.04, 1.08)	<0.001
Polyposis (None, ref)			
Yes	0.59	(0.17, 2.05)	0.4
Side (Left, ref)			
Right	1.19	(0.76, 1.87)	0.44
Neoadjuvant therapy (No, ref)			
Yes	1.5	(0.57, 4.01)	0.41
Microscopic Tumor Perforation (None, ref)			
Yes	1.75	(0.73,4.18)	0.21
Mucin (None, ref)			
Yes	1.18	(0.78, 1.80)	0.43
Gross Perforation (None, ref)			
Yes	1.34	(0.73, 2.45)	0.34
Grade (Moderate, ref)			
Poor	1.32	(0.71, 2.42)	0.38
Undifferentiated	1.4	(0.69, 2.82)	0.35
Well	1.69	(0.55, 5.16)	0.36
Margins (None, ref)			
Yes	1.67	(0.88, 3.20)	0.12
Lymphovascular invasion (None, ref)			
Yes	1.46	(0.92, 2.31)	0.1
Perineural invasion (None, ref)			
Yes	0.71	(0.40, 1.27)	0.25
Tumor Deposit (None, ref)			
Yes	1.38	(0.84, 2.28)	0.21
Stage (0, ref)			
1	1.35	(0.84, 2.16)	0.21
2	1.61	(0.91, 2.86)	0.1
3	0	(0.00, Inf)	1
Microsatellite instability (No, ref)			
Yes	0.7	(0.41, 1.18)	0.18

## Data Availability

Data available upon request to corresponding author.
